# Peri-implantitis biofilm from explanted implants in Korean patients: microbial and functional profiling

**DOI:** 10.3389/fcimb.2026.1768841

**Published:** 2026-02-06

**Authors:** Je-Hyun Eom, Mu-Yeol Cho, Ji-Won Kim, Yunwoo Kim, Seung-Jo Yang, Jiyoung Hwang, Dahye Lee, Hye-Sung Kim, Hanseung Baek, Young-Youn Kim

**Affiliations:** 1 Apple Tree Medical Foundation, Apple Tree Institute of Biomedical Science, Goyang-si, Republic of Korea; 2 Research and Development Department, DOCSmedi Co., Ltd., Goyang-si, Republic of Korea

**Keywords:** 16S rRNA sequencing, implant surface biofilm, microbial dysbiosis, oral microbiome, peri-implantitis

## Abstract

Peri-implantitis is an inflammatory disease affecting tissues surrounding dental implants, with microbial biofilms recognized as the primary etiological factor. However, most previous studies analyzed samples from peri-implant pockets, and research on biofilms directly attached to explanted implant surfaces remains limited. This study compared the microbial composition and functional characteristics of biofilms from explanted implant surfaces in peri-implantitis cases with subgingival plaque from healthy controls. A total of 41 samples (peri-implantitis n=19, healthy controls n=22) were obtained from the Apple Tree Oral Biobank. The V3-V4 region of 16S rRNA gene was sequenced using Illumina MiSeq, ASVs were generated using DADA2, and taxonomic assignment was performed using SILVA database (v138.1). Alpha and beta diversity analyses were conducted, and functional potential was predicted using PICRUSt2. The peri-implantitis group showed significantly higher Simpson index (p=0.0086) and phylogenetic diversity (p<0.0001), with distinct clustering separation between groups. Beyond well-known periodontal pathogens (Porphyromonas gingivalis, Tannerella forsythia, Treponema denticola, Filifactor alocis), the peri-implantitis group exhibited significant increases in sulfate-reducing bacteria (Desulfobulbus, Desulfovibrio) and emerging pathogens ([Eubacterium] nodatum group, [Eubacterium] saphenum group, Phocaeicola abscessus, Pseudoramibacter alactolyticus, Pyramidobacter). Health-associated bacteria (Corynebacterium, Neisseria, Capnocytophaga, Lautropia) were decreased. Functional analysis revealed enrichment in LPS biosynthesis, sulfur metabolism, iron acquisition, and amino acid degradation pathways, while carbohydrate metabolism was decreased. This study demonstrates that diverse emerging pathogens, including sulfate-reducing bacteria, are associated with peri-implantitis biofilms in explanted implant surface biofilms, contributing to expanded understanding of peri-implantitis etiology and development of candidate biomarkers.

## 1 Introduction

Dental implants are widely recognized as the highly successful and effective treatment modality for replacing missing teeth, and their use has increased dramatically over the past several decades (Giok et al., 2024). However, along with the widespread use of implants, the occurrence of peri-implant diseases has also risen, leading to growing concern. Peri-implantitis is characterized by inflammation of the peri-implant mucosa accompanied by progressive loss of supporting bone, which can ultimately lead to implant failure if left untreated (Joshi et al., 2025). Recent meta-analyses have reported the prevalence of peri-implantitis to be approximately 22% at the patient level and 9-14% at the implant level, posing a substantial clinical burden (Diaz et al., 2022; Galarraga-Vinueza et al., 2025). The development of peri-implantitis involves various risk factors beyond microbiological factors, including smoking, diabetes, history of periodontitis, poor oral hygiene, and advanced age (Cui et al., 2025). The etiology of peri-implantitis is known to be initiated by microbial biofilms, similar to periodontitis around natural teeth. Early studies reported similarities between the microbial communities of peri-implantitis and periodontitis, with the "Red complex" consisting of Porphyromonas gingivalis, Tannerella forsythia, and Treponema denticola, along with the "Orange complex" including Fusobacterium nucleatum and Prevotella intermedia, identified as major pathogens in both diseases (Chun Giok and Menon, 2023). However, with advances in next-generation sequencing (NGS) technology and deepening understanding of the oral microbiome, recent studies have reported that differences may exist between the microbial communities of peri-implantitis and periodontitis (Yu et al., 2024). In particular, implant surfaces differ from natural tooth surfaces in their physicochemical properties, and these differences may influence biofilm formation and microbial community structure. The roughness, surface energy, and chemical composition of titanium implant surfaces are known to affect initial bacterial attachment and biofilm maturation processes (Sinjab et al., 2024). Additionally, peri-implant tissues differ anatomically and immunologically from periodontal tissues around natural teeth, potentially resulting in different host-microbial interaction patterns (Di Spirito et al., 2024). For these reasons, applying the microbiological characteristics of periodontitis to peri-implantitis may have limitations. Most existing microbiome studies of peri-implantitis have been conducted by collecting samples from peri-implant pockets using paper points or curettes (Anitua et al., 2024; Anuntakarun et al., 2025). This approach has the limitation of potentially capturing more planktonic bacteria within the pocket or bacteria from soft tissue surfaces rather than biofilms directly attached to the implant surface. To accurately understand the characteristics of pathogenic biofilms that actually cause implant failure, analyzing biofilms directly attached to explanted implant surfaces may be more appropriate; however, such studies have been conducted very limitedly due to difficulties in sample acquisition. Meanwhile, recent studies have focused attention on the role of novel pathogens in peri-implantitis beyond traditionally known periodontal pathogens. Sulfate-reducing bacteria such as Desulfobulbus and Desulfovibrio can produce hydrogen sulfide, inducing tissue toxicity and inflammation, and associations with periodontitis and peri-implantitis have been reported in some studies (Cross et al., 2018). Additionally, Filifactor alocis, [Eubacterium] nodatum group, [Eubacterium] saphenum group, and Pseudoramibacter alactolyticus are emerging pathogens whose importance in periodontal disease has recently been highlighted, requiring further investigation of their role in peri-implantitis (Antezack et al., 2023; Lafaurie et al., 2023). Understanding the functional characteristics of microbial communities is also important for elucidating pathogenic mechanisms. Using functional prediction tools such as PICRUSt2 (Phylogenetic Investigation of Communities by Reconstruction of Unobserved States), the metabolic potential of microbial communities can be inferred from 16S rRNA gene data. This approach allows identification of metabolic pathways activated in disease states beyond simple taxonomic compositional changes, which can contribute to discovering novel therapeutic targets. Therefore, this study aimed to compare microbial communities of biofilms collected from explanted implant surfaces due to peri-implantitis with subgingival plaque from healthy controls through 16S rRNA gene sequencing, and to identify differences in functional characteristics using PICRUSt2 (Douglas et al., 2020). Through this approach, we sought to expand understanding of microbiological factors involved in the etiology of peri-implantitis and to explore potential potential candidate and therapeutic targets.

## 2 Materials and methods

### 2.1 Study subjects and sample collection

This study was conducted using a total of 41 plaque samples obtained from the Apple Tree Biobank of Oral-derived Specimens (Apple Tree Oral Biobank). Samples were classified into two groups: the peri-implantitis group (n=19) and the healthy control group (n=22). The overall study workflow is illustrated in **Supplementary Figure S2**.

Samples in the peri-implantitis group consisted of subgingival biofilms collected directly from entire explanted dental implant fixtures that were removed due to implant failure and donated to the Apple Tree Oral Biobank. Upon retrieval from the biobank, biofilms attached to the entire implant fixture surfaces were mechanically scraped using sterile instruments under aseptic conditions in a biosafety cabinet. All biofilm material from each explanted implant was pooled for subsequent DNA extraction.

Samples in the healthy control group consisted of subgingival plaque collected using sterile Gracey curettes (Hu-Friedy, Chicago, IL, USA) from natural teeth of systemically and periodontally healthy females aged 20 years or older. All sample collections were performed by trained dental hygienists following standardized protocols established by the Apple Tree Oral Biobank.

We acknowledge that this represents a major methodological limitation, as implant and tooth surfaces differ substantially in physicochemical properties (titanium oxide vs enamel/cementum), surface roughness, wettability, and surface energy—all factors that profoundly influence microbial adhesion and biofilm architecture independently of disease status. Ideally, subgingival biofilm from healthy, functional implants would serve as a more appropriate control to isolate disease-specific effects; however, such samples were not available in the Apple Tree Oral Biobank at the time of study design due to the ethical and practical difficulties of obtaining biofilm from healthy implants in asymptomatic patients.

All samples were stored at -80°C immediately after collection until DNA extraction. Demographic characteristics of the subjects are presented in **Table 1**. This study was approved by the Institutional Review Board of Apple Tree Medical Foundation (IRB No. ATDH-2024-0006). All participants or sample donors provided written informed consent for sample collection, storage in the biobank, and use for research purposes.

**Table 1:** Demographic characteristics of study subjects.

Characteristics	Healthy (n=22)	Peri-implantitis (n=19)
Age (years, mean ± SD)	28.5 ± 4.3	62.3 ± 11.5
Sex (Female/Male)	22/0	8/11
Smoking status		
Non-smoker	22	4
Smoker	0	9
Unknown	0	6

This study was approved by the Institutional Review Board of Apple Tree Medical Foundation (IRB No. ATDH-2024-0006).

### 2.2 DNA extraction and 16S rRNA gene sequencing

Total genomic DNA was extracted from plaque samples using the LaboPass™ Bacteria Genomic DNA Isolation Kit Mini (Cat. No. CMBA0112, Cosmogenetech, Seoul, Korea) following the manufacturer's protocol for Gram-negative bacteria. The V3-V4 hypervariable region of the 16S rRNA gene was amplified using primer pair 341F (5'-CCTACGGGNGGCWGCAG-3') and 805R (5'-GACTACHVGGGTATCTAATCC-3'). Paired-end sequencing (2 × 300 bp) was performed on the Illumina MiSeq platform.

### 2.3 Quality control

Sequencing runs included negative extraction controls (reagent-only controls processed through the entire DNA extraction protocol) and PCR negative controls (no-template controls) to monitor for contamination. No amplification was detected in any negative controls, confirming absence of contamination. Mock community controls were not included in this study, as ASV generation accuracy has been extensively validated for the DADA2 pipeline in previous studies. Samples with <10, 000 reads after quality filtering were excluded (n=2 samples excluded).

### 2.4 Data preprocessing and ASV GENERATION

Following sequencing, adapter and primer sequences were removed from raw data using Cutadapt (v3.2). For generation of Amplicon Sequence Variants (ASVs), the DADA2 pipeline (v1.30.0) was used in the R (v4.3.3) environment. Forward and reverse reads were truncated at 250 bp and 200 bp, respectively, based on quality score profiles showing median Q-scores >30 up to these positions, and sequences with expected errors greater than 2 were excluded. These truncation lengths were selected to: (1) maximize read quality (maintaining Q >30), (2) ensure sufficient overlap for reliable paired-end merging (expected overlap ~50 bp for V3-V4 amplicons), and (3) retain phylogenetic information across the full V3-V4 region. Erroneous reads were denoised based on the constructed error model, and paired-end reads were merged based on overlapping regions after error correction. Chimeric sequences were removed using the consensus method with the removeBimeraDenovo function in DADA2. ASVs shorter than 350 bp were filtered, and a final set of 2, 997 ASVs was used for downstream analysis. To normalize for differences in sequencing depth across samples, rarefaction was performed based on the minimum sequencing depth of 24, 639 reads.

### 2.5 Taxonomic assignment

Taxonomic classification was performed using the assignTaxonomy function in DADA2 with the SILVA reference database (v138.1) at a minimum bootstrap confidence of 50%. This threshold was selected as a balance between taxonomic assignment confidence and genus-level classification coverage, consistent with DADA2 developer recommendations for 16S rRNA studies. Higher thresholds (e.g., 80%) resulted in excessive unassigned taxa at genus level without improving overall classification accuracy in preliminary analyses. Species-level classification was performed using the addSpecies function with the SILVA species assignment database (v138.1).

### 2.6 Alpha and beta diversity analysis

Alpha diversity metrics including observed ASVs, Shannon index, Simpson index, and phylogenetic diversity (PD whole tree) were calculated using the vegan (v2.6) and picante (v1.8) packages in R. For phylogenetic diversity analysis, ASV sequences were aligned using MAFFT (v7.525), and a phylogenetic tree was constructed using FastTree (v2.2.0) with the generalized time-reversible (GTR) model. Differences in alpha diversity metrics between groups were assessed using the Mann-Whitney U test.

Beta diversity was assessed using Bray-Curtis dissimilarity. Principal Coordinates Analysis (PCoA) was performed to visualize differences in community structure between groups. Statistical significance of differences in community composition was evaluated using Permutational Multivariate Analysis of Variance (PERMANOVA) with 999 permutations.

### 2.7 Differential abundance analysis

Differential abundance of bacterial taxa between the peri-implantitis and healthy control groups was assessed using the Mann-Whitney U test. Linear Discriminant Analysis Effect Size (LEfSe) analysis was performed to identify taxa showing significant differential abundance. A p-value < 0.05 was considered statistically significant.

### 2.8 Functional prediction

The functional potential of microbial communities was predicted using PICRUSt2 (Phylogenetic Investigation of Communities by Reconstruction of Unobserved States, v2.5.2). ASV sequences were placed into the reference tree using EPA-NG, and gene family abundance was inferred based on Kyoto Encyclopedia of Genes and Genomes (KEGG) Orthologs (KOs). MetaCyc pathway abundance was predicted from enzyme abundance.

It is important to note that PICRUSt2 provides inferred functional potential based on 16S rRNA gene sequences and reference genome databases, rather than measuring actual gene expression or metabolic activity. Prediction quality was assessed using Nearest Sequenced Taxon Index (NSTI) values, with mean NSTI < 0.15 indicating high prediction confidence for our dataset. Differential pathway analysis between groups was performed using the Mann-Whitney U test with FDR correction.

### 2.9 Statistical analysis

Statistical analyses were performed using the scipy (v1.11), pandas (v2.0), and scikit-learn (v1.3) libraries in Python (v3.10), and vegan (v2.6) and picante (v1.8) packages in R (v4.3.3). Data visualization was performed using matplotlib (v3.7), seaborn (v0.12), and ggplot2 (v3.4.0). All statistical tests were two-tailed. The Benjamini-Hochberg false discovery rate (FDR) method was applied to control for multiple testing, with statistical significance defined as FDR-adjusted p < 0.05. For LEfSe analysis, Kruskal-Wallis test (alpha = 0.05) and Linear Discriminant Analysis (LDA score threshold = 2.0) were applied. After FDR correction, 63 of 182 tested genera remained significant (**Supplementary Table S2**), and all 68 LEfSe-identified features remained significant (**Supplementary Table S3**), with a comprehensive multiple testing correction summary provided in **Supplementary Table S4**. Rarefaction to the minimum sequencing depth (24, 639 reads) was performed to normalize for sequencing depth differences. To assess robustness to smoking confounding, sensitivity analysis was performed comparing non-smoking individuals only (Healthy: n=22 vs Peri-implantitis: n=4), with results in **Supplementary Figure S1** and **Supplementary Table S1**.

## 3 Results

### 3.1 Alpha diversity analysis

**Figure 1:**
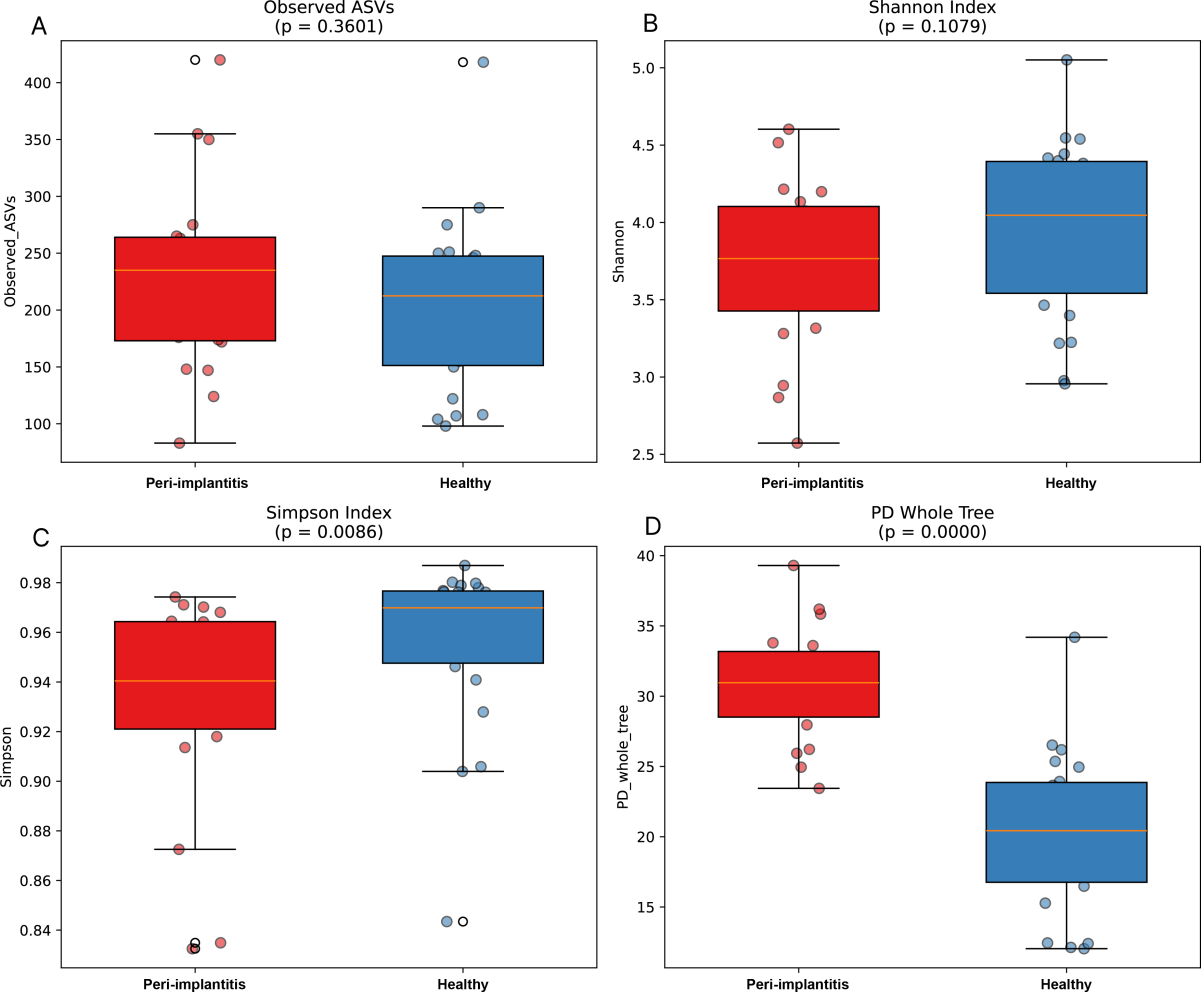
Alpha diversity comparison between peri-implantitis and healthy control groups. Comparison of alpha diversity metrics between the peri-implantitis group (red, n=19) and healthy control group (blue, n=22). **(A)** Observed ASVs, **(B)** Shannon index, **(C)** Simpson index, **(D)** Phylogenetic diversity (PD whole tree). Box plots represent median and interquartile range, and dots represent individual samples. Differences between groups were assessed using the Mann-Whitney U test. Observed ASVs (p = 0.3601) and Shannon index (p = 0.1079) showed no significant differences between the two groups, while Simpson index (p = 0.0086) and PD whole tree (p < 0.0001) showed significant differences.

Four alpha diversity metrics were analyzed to compare microbial community diversity between the peri-implantitis and healthy control groups (**Figure 1**). Observed ASVs showed no significant difference between the peri-implantitis and healthy control groups (p = 0.3601), and the Shannon index also showed no significant difference between the two groups (p = 0.1079). However, the Simpson index was significantly higher in the healthy control group compared to the peri-implantitis group (p = 0.0086), indicating greater evenness in the healthy group. Phylogenetic diversity reflected by PD whole tree was significantly higher in the peri-implantitis group compared to the healthy control group (p < 0.0001). These results suggest that phylogenetically diverse bacterial species were introduced in the peri-implantitis group.

### 3.2 Beta diversity analysis

**Figure 2:**
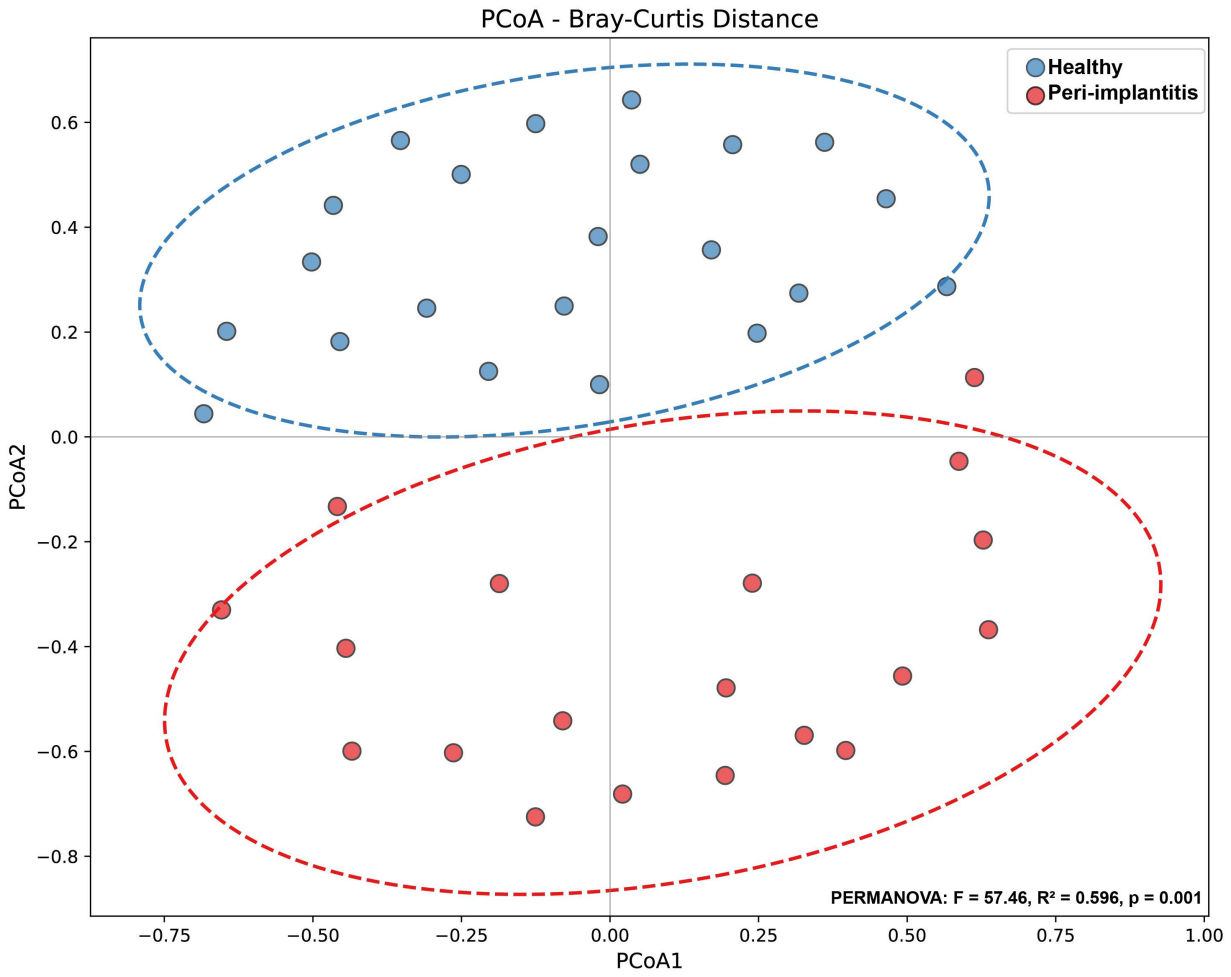
Principal Coordinates Analysis (PCoA) based on Bray-Curtis distance. Principal Coordinates Analysis (PCoA) results based on Bray-Curtis dissimilarity. Each point represents an individual sample, with blue indicating the healthy control group (n=22) and red indicating the peri-implantitis group (n=19). Dashed ellipses represent 95% confidence intervals for each group. Differences in microbial community structure between the two groups were assessed using PERMANOVA (F = 57.46, R² = 0.596, p = 0.001, 999 permutations).

Principal Coordinates Analysis (PCoA) based on Bray-Curtis distance was performed to visualize differences in microbial community structure between the two groups (**Figure 2**). In the PCoA plot, the peri-implantitis and healthy control groups formed distinctly separated clusters. PERMANOVA analysis revealed that the difference in microbial community composition between the two groups was highly statistically significant (F = 57.46, R² = 0.596, p = 0.001), with the group variable explaining approximately 59.6% of the total community variation. This strongly suggests that the microbial communities of peri-implantitis and healthy states have fundamentally different structures.

### 3.3 Sensitivity analysis: non-smokers only

To address potential confounding effects of smoking on microbiome composition, we performed a sensitivity analysis comparing only non-smoking individuals (Healthy: n=22, all non-smokers vs Peri-implantitis: n=4, confirmed non-smokers). Despite the limited sample size of non-smoking peri-implantitis cases, several key findings emerged:Beta diversity analysis revealed persistent significant differentiation between groups (PERMANOVA: R²=0.177, p=0.001), although with reduced effect size compared to the full dataset (R²=0.596). Principal coordinates analysis showed clear clustering separation with minimal overlap between groups (**Supplementary Figure S1A**).

Alpha diversity showed significant differences in Shannon diversity (p=0.048) and Simpson index (p=0.040), with healthy controls maintaining higher diversity indices (**Supplementary Figure S1B**). Key pathogenic genera remained significantly enriched in non-smoking peri-implantitis cases, including Pyramidobacter (p<0.001), Porphyromonas (p=0.001), Phocaeicola (p=0.003), Tannerella (p=0.02), and Treponema (p=0.02), following FDR correction for multiple testing (**Supplementary Figure S1C**; **Supplementary Table S1**). These exploratory findings suggest that disease-associated microbial dysbiosis persists even when smoking is controlled, supporting a genuine association between the identified microbial profiles and peri-implantitis beyond smoking effects alone. However, the small sample size (n=4 non-smoking peri-implantitis cases) substantially limits statistical power and prevents definitive conclusions. The reduced effect size (R²=0.177 vs 0.596) indicates that demographic factors including smoking contribute meaningfully to microbiome variation.

### 3.4 Taxonomic composition

#### 3.4.1 Phylum level composition

**Figure 3:**
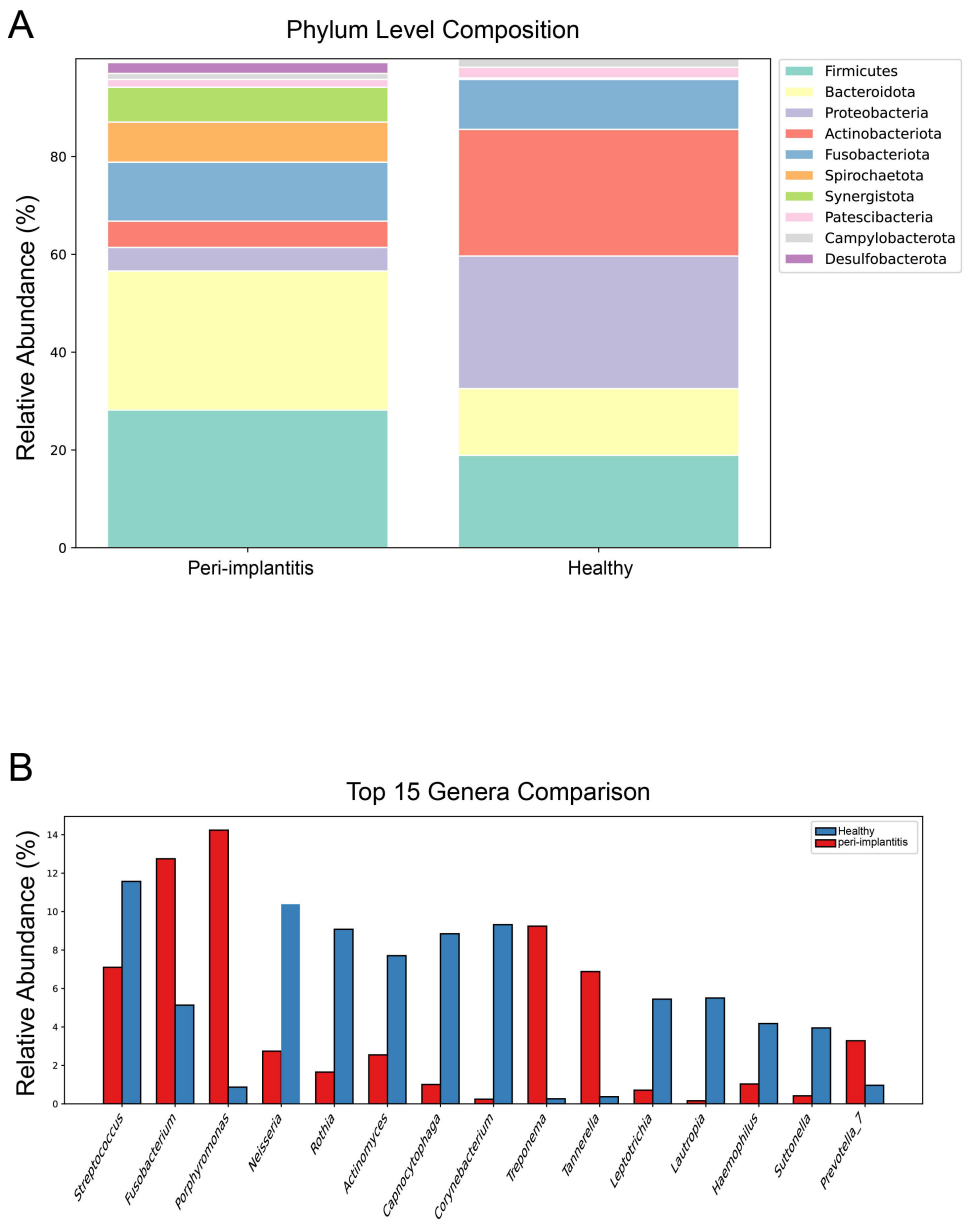
Taxonomic composition comparison between peri-implantitis and healthy control groups. **(A)** Microbial composition at the phylum level. Relative abundance of the top 10 phyla is shown as stacked bar graphs. **(B)** Comparison of the top 15 taxa at the genus level. Bar graphs represent the mean relative abundance (%) of each genus, with blue indicating the healthy control group and red indicating the peri-implantitis group.

Microbial composition at the phylum level was compared between the two groups (**Figure 3A**). In both groups, Firmicutes, Bacteroidota, Proteobacteria, Actinobacteriota, and Fusobacteriota were identified as dominant phyla. In the peri-implantitis group, the relative abundance of Spirochaetota and Synergistota increased compared to the healthy control group, while Actinobacteriota and Proteobacteria showed a decreasing trend. Notably, Desulfobacterota, which includes sulfate-reducing bacteria, was observed in the peri-implantitis group but was rarely detected in the healthy control group.

#### 3.4.2 Genus level composition

The relative abundance of the top 15 taxa at the genus level was compared (**Figure 3B**). In the healthy control group, health-associated bacteria such as Streptococcus, Neisseria, Rothia, Actinomyces, and Capnocytophaga were predominant. In contrast, the peri-implantitis group showed significantly increased relative abundance of pathogenic bacteria including Porphyromonas, Fusobacterium, Treponema, and Tannerella. Streptococcus was the most abundant genus in both groups, but accounted for a higher proportion in the healthy control group. Individual sample microbial compositions are presented in **Supplementary Figure S3**, and heatmap analysis results of the top 30 genera are presented in **Supplementary Figure S4**.

### 3.5 Differential abundance analysis

#### 3.5.1 LEfSe analysis

**Figure 4:**
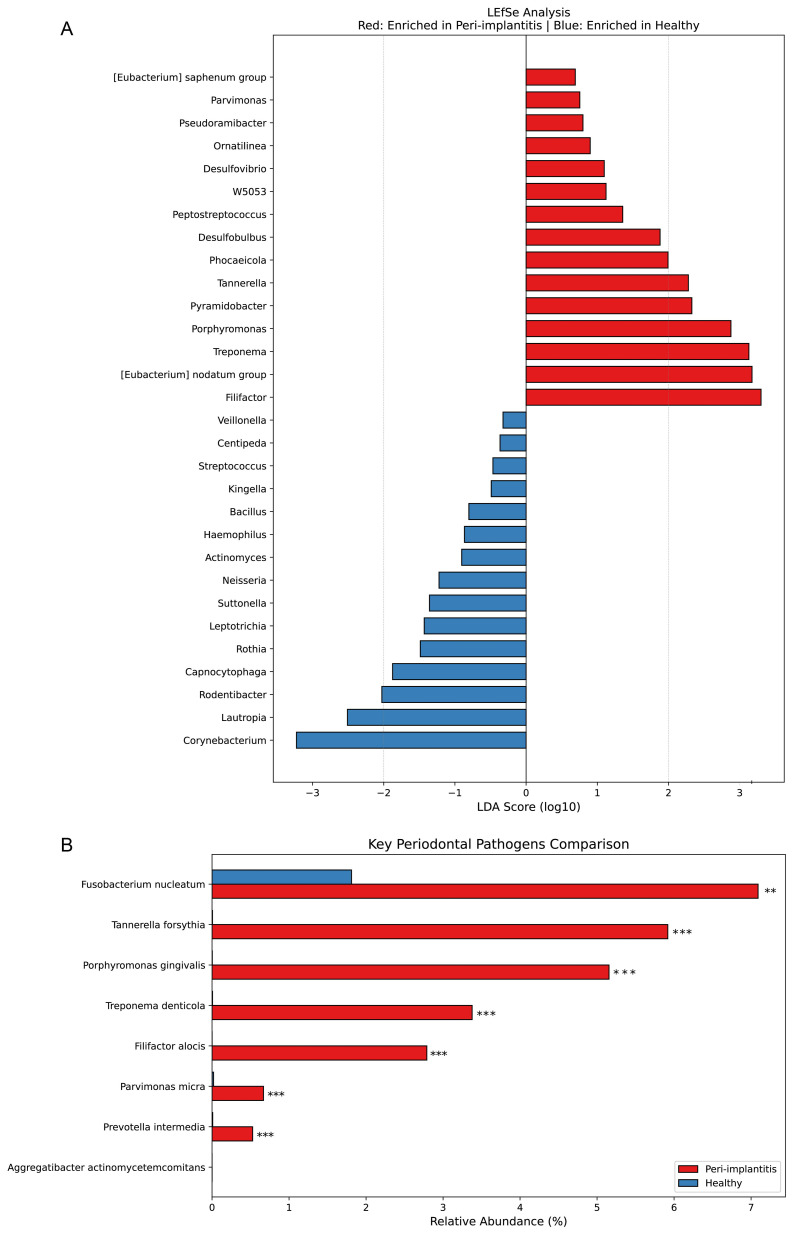
Differential abundance analysis: LEfSe analysis and comparison of key periodontal pathogens. **(A)** Linear Discriminant Analysis Effect Size (LEfSe) analysis results. Genera with positive LDA scores (log10, red bars) represent taxa enriched in the peri-implantitis group, while those with negative scores (blue bars) represent taxa enriched in the healthy control group. Kruskal-Wallis test with alpha = 0.05 and LDA score threshold = 2.0 were applied. All 68 identified features remained significant after FDR correction. **(B)** Comparison of relative abundance of well-known key periodontal pathogens. Bar graphs represent mean relative abundance (%), with red indicating the peri-implantitis group and blue indicating the healthy control group. Statistical significance assessed using Mann-Whitney U test with Benjamini-Hochberg FDR correction. **FDR-adjusted p < 0.01, ***FDR-adjusted p < 0.001.

Linear Discriminant Analysis Effect Size (LEfSe) analysis was performed to identify significantly differentially abundant taxa between the two groups (**Figure 4A**). Genera significantly increased in the peri-implantitis group included Filifactor (LDA score > 3), [Eubacterium] nodatum group, Treponema, Porphyromonas, Pyramidobacter, Tannerella, Phocaeicola, Desulfobulbus, Peptostreptococcus, and Desulfovibrio. In contrast, the healthy control group showed significant increases in Corynebacterium, Lautropia, Capnocytophaga, Rothia, Neisseria, Actinomyces, and Streptococcus. Complete LEfSe results with FDR-adjusted p-values are provided in **Supplementary Table S3**.

#### 3.5.2 Key periodontal pathogens

Comparison of the relative abundance of well-known periodontal pathogens revealed (**Figure 4B**) that Fusobacterium nucleatum, Tannerella forsythia, Porphyromonas gingivalis, Treponema denticola, Filifactor alocis, Parvimonas micra, and Prevotella intermedia all showed significantly higher abundance in the peri-implantitis group compared to the healthy control group (p < 0.01 or p < 0.001). Particularly, the Red complex pathogens P. gingivalis, T. forsythia, and T. denticola were markedly elevated in the peri-implantitis group. Aggregatibacter actinomycetemcomitans showed very low abundance in both groups.

#### 3.5.3 Differentially abundant species

**Figure 5:**
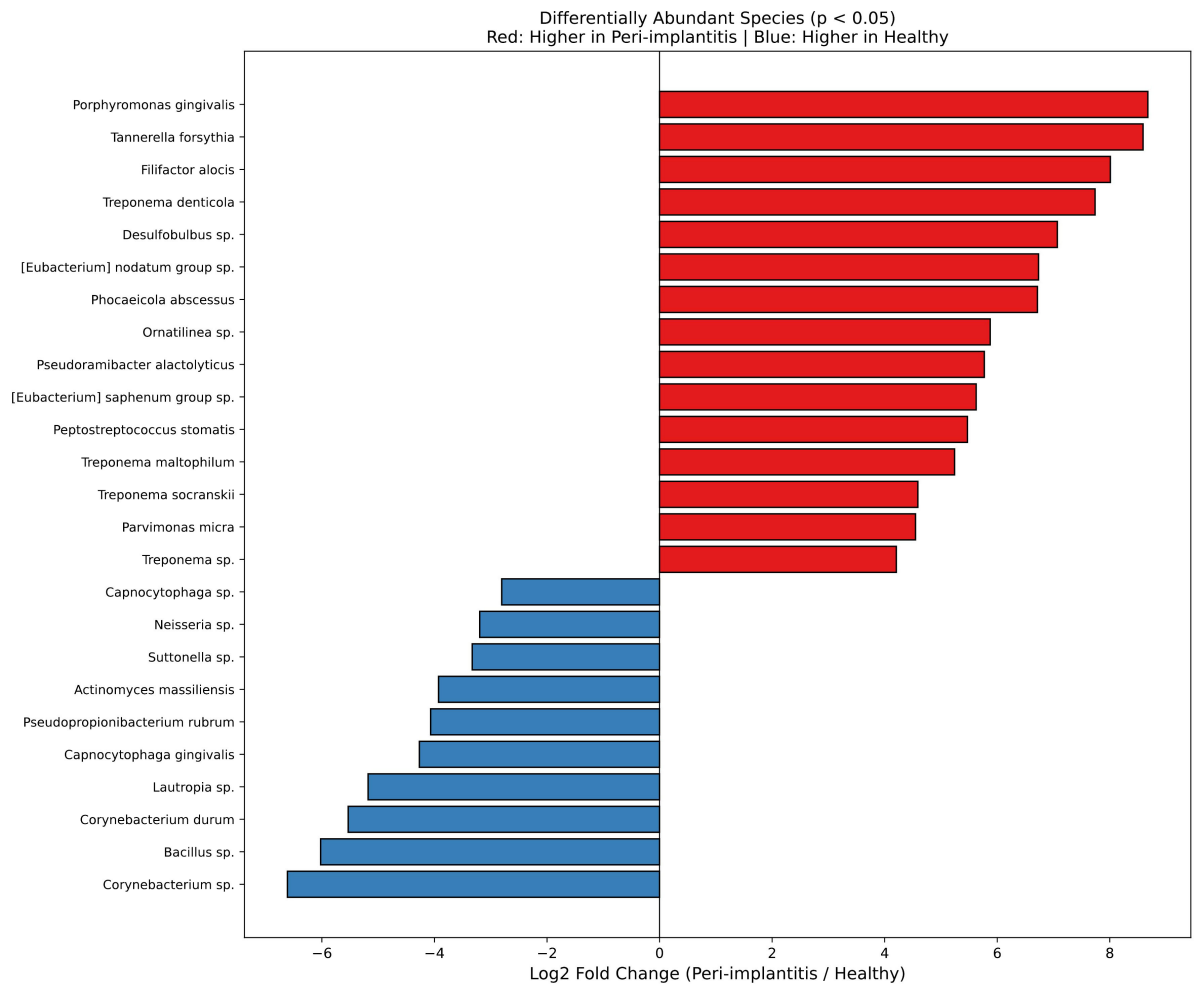
Species-level differential abundance analysis. Significantly differentially abundant species between the peri-implantitis and healthy control groups (FDR-adjusted p < 0.05). Bar graphs represent Log2 fold change (peri-implantitis/healthy control). Positive values (red bars) indicate species increased in the peri-implantitis group, while negative values (blue bars) indicate species increased in the healthy control group. Statistical significance: Mann-Whitney U test, FDR-adjusted p < 0.05.

Species-level differential abundance analysis revealed (**Figure 5**) that species significantly increased in the peri-implantitis group included P. gingivalis (Log2FC ≈ 8), T. forsythia, F. alocis, T. denticola, Desulfobulbus sp., [Eubacterium] nodatum group sp., Phocaeicola abscessus, Ornatilinea sp., Pseudoramibacter alactolyticus, [Eubacterium] saphenum group sp., Peptostreptococcus stomatis, Treponema maltophilum, T. socranskii, and Parvimonas micra. Notably, in addition to well-known periodontal pathogens, emerging pathogens such as the sulfate-reducing bacterium Desulfobulbus sp. and [Eubacterium] nodatum group and [Eubacterium] saphenum group were significantly increased in the peri-implantitis group. Species increased in the healthy control group included health-associated bacteria such as Corynebacterium sp. (Log2FC ≈ -6), Bacillus sp., Corynebacterium durum, Lautropia sp., Capnocytophaga gingivalis, Neisseria sp., and Actinomyces massiliensis. Genus-level differential abundance analysis results are presented in **Supplementary Figure S5**. Complete genus-level differential abundance results with FDR correction are provided in **Supplementary Table S****2**.

### 3.6 Functional prediction analysis

#### 3.6.1 Key functional categories

**Figure 6:**
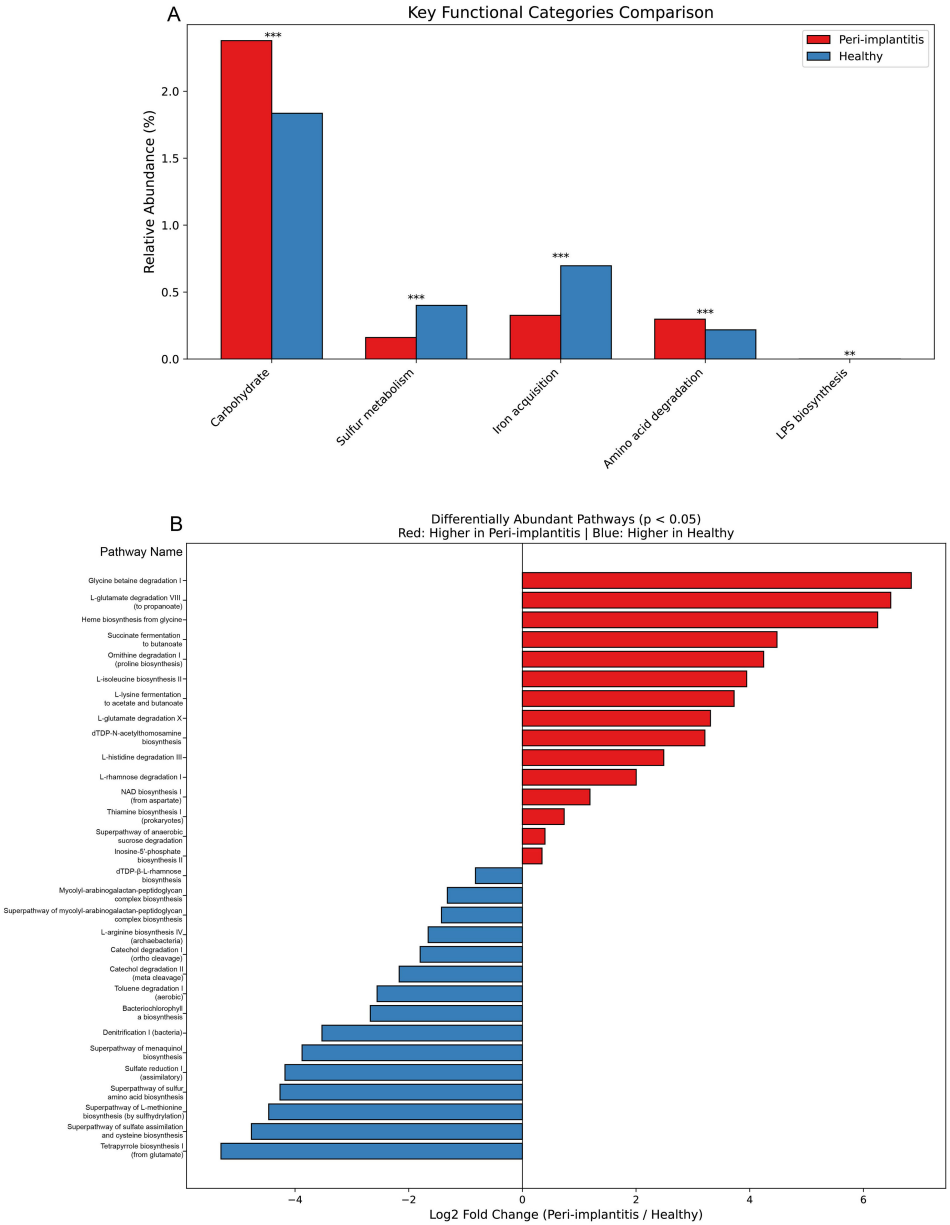
Functional prediction analysis of microbial communities. Predicted functional potential inferred from 16S rRNA gene data using PICRUSt2. **(A)** Predicted relative abundance of major functional categories. Bar graphs represent mean predicted abundance (%), with red indicating peri-implantitis and blue indicating healthy controls. Statistical significance: Mann-Whitney U test with Benjamini-Hochberg FDR correction. **FDR q < 0.01, ***FDR q < 0.001. **(B)** Predicted differential pathway enrichment at the MetaCyc pathway level. Bar graphs show Log2 fold change (peri-implantitis/healthy). Positive values (red bars) indicate predicted enrichment in peri-implantitis, while negative values (blue bars) indicate predicted enrichment in healthy controls (FDR q < 0.05). These represent predicted metabolic capabilities based on taxonomic composition, not measured gene expression or activity.

The functional potential of microbial communities was predicted using PICRUSt2, and major functional categories were compared (**Figure 6A**). Pathways related to carbohydrate metabolism showed predicted higher abundance in the healthy control group compared to the peri-implantitis group (FDR q < 0.001). In contrast, the peri-implantitis group showed predicted enrichment of pathways related to sulfur metabolism (FDR q < 0.001), iron acquisition (FDR q < 0.001), amino acid degradation (FDR q < 0.001), and LPS biosynthesis (FDR q < 0.01). These predicted pathway enrichments may suggest potential environmental changes associated with pathogenic conditions in peri-implantitis.

#### 3.6.2 Differentially abundant pathways

Differential abundance analysis was performed at the MetaCyc pathway level (**Figure 6B**). Pathways showing predicted higher abundance in the peri-implantitis group included amino acid degradation and fermentation-related pathways such as glycine betaine degradation I, L-glutamate degradation VIII (to propanoate), heme biosynthesis from glycine, succinate fermentation to butanoate, and L-lysine fermentation to acetate and butanoate (FDR-adjusted p < 0.05). The predicted functional potential may suggest capacity for tissue destruction and inflammatory responses through protein degradation and short-chain fatty acid production.

Pathways showing predicted higher abundance in the healthy control group included tetrapyrrole biosynthesis I, superpathway of sulfate assimilation and cysteine biosynthesis, superpathway of L-methionine biosynthesis, sulfate reduction I (assimilatory), denitrification I (bacteria), and catechol degradation I/II (FDR-adjusted p < 0.05). These findings suggest that assimilatory sulfur metabolism and aerobic degradation pathways may be predominant in healthy oral environments.

## 4 Discussion

This study analyzed the microbial community of biofilms collected directly from explanted implant surfaces due to peri-implantitis through 16S rRNA gene sequencing and compared them with subgingival plaque from healthy controls. The results revealed distinct differences in microbial community structure between the peri-implantitis and healthy control groups, confirming that sulfate-reducing bacteria and various emerging pathogens, in addition to well-known periodontal pathogens, are associated with peri-implantitis. Additionally, functional prediction analysis identified predicted metabolic pathway enrichment in peri-implantitis.

Comparison with Previous Studies.

The microbial profile associated with peri-implantitis identified in this study was largely consistent with previous research findings. The Red complex, consisting of Porphyromonas gingivalis, Tannerella forsythia, and Treponema denticola, has been reported as a core pathogen not only in periodontitis but also in peri-implantitis, and these bacteria were significantly increased in the peri-implantitis group in this study (Fernandes et al., 2024). Filifactor alocis has recently received attention as an important pathogen in periodontal disease, and in this study, it showed the highest LDA score in the peri-implantitis group, suggesting its potential as a strong biomarker (Antezack et al., 2023).

Health-associated bacteria predominant in the healthy control group, including Streptococcus, Neisseria, Rothia, Actinomyces, and Corynebacterium, are commensal bacteria commonly observed in healthy oral environments, consistent with previous studies (Anuntakarun et al., 2025; Bessa et al., 2025). These bacteria are known to contribute to maintaining oral homeostasis and play a role in inhibiting colonization by pathogenic bacteria.

The high R² value (0.596) observed in beta diversity analysis indicates that the difference in microbial community between peri-implantitis and healthy states is very large, which is similar to or higher than values reported in previous periodontitis studies (Joshi et al., 2025). These results support that peri-implantitis is a disease with distinct microbiological characteristics.

Novel Findings of This Study

This study presents novel findings that differentiate it from previous research in several aspects.

First, biofilms were collected and analyzed directly from explanted implant surfaces. Most previous studies collected samples from peri-implant pockets using paper points or curettes, which may reflect more planktonic bacteria within the pocket rather than biofilms directly attached to the implant surface (Faveri et al., 2014). The explanted implant surface biofilms used in this study have the advantage of more accurately reflecting the pathogenic community that actually caused implant failure.

Second, we confirmed that sulfate-reducing bacteria (SRB), Desulfobulbus and Desulfovibrio, were significantly associated with peri-implantitis. Sulfate-reducing bacteria can contribute to cytotoxicity, inflammation induction, and tissue destruction through the production of hydrogen sulfide (H_2_S) (Kushkevych et al., 2020). Hydrogen sulfide is known to inhibit mitochondrial function, damage epithelial barriers, and promote inflammatory cytokine secretion. Although some studies have reported associations between periodontitis and sulfate-reducing bacteria, their role in peri-implantitis has not yet been sufficiently elucidated. Our findings suggest that sulfate-reducing bacteria may contribute to the etiology of peri-implantitis.

Third, emerging pathogens including [Eubacterium] nodatum group, [Eubacterium] saphenum group, Phocaeicola abscessus, Pseudoramibacter alactolyticus, and Pyramidobacter were significantly increased in the peri-implantitis group (Antezack et al., 2023). Although associations of these bacteria with periodontal disease have recently been reported, their role in peri-implantitis has been studied limitedly. Particularly, [Eubacterium] nodatum group and [Eubacterium] saphenum group are known to proliferate in anaerobic environments and possess proteolytic activity, potentially contributing to destruction of peri-implant tissues.

Fourth, predicted metabolic pathway enrichment in peri-implantitis were identified through functional prediction analysis using PICRUSt2. The pattern of increased pathways related to amino acid degradation, sulfur metabolism, iron acquisition, and LPS biosynthesis, along with decreased carbohydrate metabolism pathways in the peri-implantitis group, reflects a shift toward a proteolytic and anaerobic environment (Song et al., 2024). These functional changes may be closely associated with the establishment of a pathogenic environment in peri-implantitis and can contribute to discovering novel therapeutic targets.

Fifth, this study was conducted on Korean patients. Most previous peri-implantitis microbiome studies have been conducted on Western patients, and research on Asian patients, particularly Korean patients, is limited. Since the oral microbiome can differ according to genetic background, dietary habits, and lifestyle, our findings can contribute to understanding peri-implantitis microbiome characteristics in Korean patients (Kim et al., 2022).

Mechanistic Interpretation

The predicted enrichment of amino acid degradation pathways (glycine betaine degradation, L-glutamate degradation, L-lysine fermentation, etc.) in the peri- implantitis group suggests potential capacity for host tissue protein degradation. Metabolic products that could potentially be generated during these processes, including ammonia, hydrogen sulfide, and short-chain fatty acids (such as butyrate), might exhibit tissue toxicity and promote inflammatory responses. While PICRUSt2 predictions provide valuable hypotheses about community functional potential, these inferences should be validated through direct measurement of metabolic activity using metatranscriptomic or metametabolomic approaches (see Limitations) (Basic and Dahlén, 2023).

The increase in sulfur metabolism pathways is consistent with the increase in sulfate-reducing bacteria, and the hydrogen sulfide they produce can be involved in various pathogenic mechanisms including cytotoxicity, DNA damage, and inflammation induction. The increase in iron acquisition pathways reflects strategies for securing iron, which is essential for pathogen survival and proliferation, and can be interpreted as an adaptive mechanism of pathogens to counteract the host's iron restriction defense mechanism (nutritional immunity). The increase in LPS biosynthesis pathways is consistent with the increase in Gram-negative pathogens, and LPS potently activates the host inflammatory response through TLR4 receptors.

In contrast, the increased carbohydrate metabolism pathways in the healthy control group reflect a healthy oral environment where saccharolytic metabolism is predominant. The increase in sulfur assimilation pathways and aerobic degradation pathways indicates that aerobic or facultative anaerobic bacteria are predominant in healthy oral environments.

Clinical Implications

Our findings have several clinical implications that warrant careful consideration. First, the characteristic microbial profiles identified in peri- implantitis represent potential candidate biomarkers that require validation before clinical implementation. Particularly, Filifactor alocis, Desulfobulbus, and [Eubacterium] nodatum group are often not included in existing periodontal pathogen tests, suggesting the need to develop expanded microbial testing panels that include these bacteria. However, diagnostic utility must be established through: (1) validation in independent cohorts with adequate sample size, (2) assessment of diagnostic performance metrics (sensitivity, specificity, ROC analysis), (3) comparison with existing diagnostic methods, and (4) evaluation of clinical utility and cost-effectiveness. Our findings provide preliminary evidence supporting these bacteria as candidate markers, but prospective validation studies are essential before clinical translation (Antezack et al., 2023).

Second, with the confirmed role of sulfate-reducing bacteria and sulfur metabolism, the possibility of developing novel therapeutic strategies targeting hydrogen sulfide production inhibition or sulfate-reducing bacteria is suggested. Third, the activation of amino acid degradation and proteolytic pathways can provide a rationale for nutritional approaches that restrict protein substrate supply in peri-implantitis patients or for developing therapeutics that inhibit these metabolic pathways.

## 4.1 Limitations

This study has several important limitations that must be carefully considered when interpreting our findings.

First and most critically, there is a fundamental difference in sample substrate between groups. The peri-implantitis group analyzed biofilms from titanium implant surfaces, while the healthy control group analyzed subgingival plaque from natural tooth surfaces. Titanium and tooth surfaces differ substantially in surface chemistry, energy, roughness, and electrical charge properties—all factors that strongly influence microbial adhesion and biofilm architecture independently of disease status. Additionally, peri-implant and periodontal tissues differ anatomically and immunologically, potentially creating different microenvironments that shape microbial communities. The observed microbial differences may therefore reflect: (1) disease-specific dysbiosis, (2) inherent surface-specific colonization patterns, or (3) a combination of both. We cannot definitively distinguish between these possibilities with our current study design. Ideally, biofilm from healthy implants would serve as the appropriate control, but such samples were not available due to ethical and practical challenges. Despite this limitation, our findings remain valuable because: (1) the identified pathogens align closely with previous peri-implantitis studies using pocket samples, (2) the strong clustering separation (R²=0.596) suggests robust disease associations, (3) our sensitivity analysis showed persistent dysbiosis independent of smoking (R²=0.177, p=0.001), and (4) predicted functional shifts align with known pathogenic mechanisms. Nevertheless, future studies using healthy implant biofilms as controls are essential for validation.

Second, substantial demographic differences exist between groups. The peri-implantitis group was older (62.3 vs 28.5 years) and predominantly male, while healthy controls were all female. Age, sex, and hormonal status influence oral microbiome composition, and these differences may contribute to observed variations. The demographic differences between groups (age, sex, smoking) are known to independently influence oral microbiome composition through multiple mechanisms. Aging is associated with decreased salivary flow, changes in immune function, and shifts toward more anaerobic communities. Sex hormones influence gingival vascularity and immune responses. Smoking creates a hypoxic environment favoring anaerobes and directly suppresses immune surveillance. When combined with different substrate properties (titanium vs tooth), these factors may interact synergistically to shape distinct microenvironments. While our sensitivity analysis suggests disease-associated dysbiosis persists independent of smoking, the reduced effect size (R²=0.177 vs 0.596) indicates that demographic factors substantially contribute to the observed variation.

Third, smoking status differed markedly. All healthy controls were non- smokers, while the peri-implantitis group included 9 smokers, 4 non- smokers, and 6 with uncertain status. Our sensitivity analysis comparing only non-smokers (n=4 peri-implantitis) showed persistent microbial differences, suggesting disease-associated dysbiosis exists independent of smoking, though the small sample size limits definitive conclusions.

Fourth, the retrospective biobank-based design precluded prospective demographic matching or multivariable adjustment for confounders.

Fifth, this cross-sectional study cannot establish causal relationships. We cannot distinguish whether microbial changes are a cause or consequence of peri-implantitis, nor can we track disease progression.

Sixth, PICRUSt2 infers functional potential from 16S rRNA sequences but does not measure actual gene expression, enzyme activity, or metabolite production. The predicted functional profiles represent potential metabolic capabilities rather than realized activities. Our findings should be interpreted as preliminary hypotheses requiring validation through metatranscriptomic or metametabolomic analysis. The NSTI values (mean < 0.15) indicate good prediction confidence, but direct validation remains essential.

Seventh, detailed implant characteristics (brand, surface topography) were unavailable due to biobank privacy policies. Implant surface heterogeneity may contribute to variability in microbial composition.

Future Research Directions

To address the limitations of this study, the following approaches are needed in future research. First, studies using control groups matched for age, sex, and smoking status are necessary. Second, longitudinal studies comparing microbial communities across stages of healthy implants, peri-implant mucositis, and peri-implantitis are needed. Third, basic research is needed to elucidate the pathogenic mechanisms of sulfate-reducing bacteria and emerging pathogens. Fourth, functional prediction results need to be validated through metatranscriptomic or metametabolomic analysis. Fifth, large-scale validation studies are needed to evaluate the diagnostic potential of the candidate biomarkers identified in this study. Specifically, future research should: (1) assess diagnostic performance (sensitivity, specificity, positive/negative predictive values) in independent cohorts, (2) determine optimal cutoff values for microbial abundance thresholds, (3) evaluate early diagnostic capability for detecting peri-implantitis before radiographic bone loss, (4) compare diagnostic accuracy with existing clinical and radiographic assessments, and (5) assess feasibility for point-of-care testing applications.

## 5 Conclusion

This study demonstrated that the microbial community of biofilms from explanted implant surfaces in peri-implantitis is distinctly different from healthy subgingival plaque, and that sulfate-reducing bacteria (Desulfobulbus, Desulfovibrio) and various emerging pathogens ([Eubacterium] nodatum group, [Eubacterium] saphenum group, Phocaeicola abscessus, Pseudoramibacter alactolyticus, etc.), in addition to known periodontal pathogens, are associated with peri- implantitis biofilms. Functional prediction analysis revealed predicted enrichment of pathways related to amino acid degradation, sulfur metabolism, iron acquisition, and LPS biosynthesis in peri-implantitis, along with predicted decreased carbohydrate metabolism. Despite the fundamental limitation of comparing biofilms from different substrates (titanium implants vs natural teeth), our findings showing strong microbial differentiation (R²=0.596) and persistent dysbiosis independent of smoking may contribute to expanding understanding of peri-implantitis etiology. The identified microbial profiles represent candidate biomarkers and potential therapeutic targets that warrant validation in independent cohorts using appropriate controls.

## Data Availability

The raw sequencing data presented in the study are deposited in the NCBI Sequence Read Archive (SRA) repository under BioProject accession number PRJNA1414202 (SRA Study: SRP666316).

## References

[B1] Anitua E. Murias-Freijo A. Tierno R. Tejero R. Alkhraisat M. H. (2024). Assessing peri-implant bacterial community structure: the effect of microbiome sample collection method. BMC Oral. Health 24, 1001. doi: 10.1186/s12903-024-04675-y, PMID: 39187802 PMC11348724

[B2] Antezack A. Etchecopar-Etchart D. La Scola B. Monnet-Corti V. (2023). New putative periodontopathogens and periodontal health-associated species: a systematic review and meta-analysis. J. Periodontal Res. 58, 893–906. doi: 10.1111/jre.13173, PMID: 37572051

[B3] Anuntakarun S. Thaweesapphithak S. Krasaesin A. Prommanee S. Arunyanak S. Kungsadalpipob K. . (2025). Microbiome Shifts in Peri-Implantitis: Longitudinal Characterization of Dysbiosis and Resolution. Int. Dental J. 75, 100951. doi: 10.1016/j.identj.2025.100951, PMID: 40811957 PMC12361765

[B4] Basic A. Dahlén G. (2023). Microbial metabolites in the pathogenesis of periodontal diseases: a narrative review. Front. Oral. Health 4, 1210200. doi: 10.3389/froh.2023.1210200, PMID: 37388417 PMC10300593

[B5] Bessa L. J. Egas C. Pires C. Proença L. Mascarenhas P. Pais R. J. . (2025). Linking peri-implantitis to microbiome changes in affected implants, healthy implants, and saliva: a cross-sectional pilot study. Front. Cell. Infection Microbiol. 15, 1543100. doi: 10.3389/fcimb.2025.1543100, PMID: 40313461 PMC12043654

[B7] Chun Giok K. Menon R. K. (2023). The microbiome of peri-implantitis: A systematic review of next-generation sequencing studies. Antibiotics 12, 1610., PMID: 37998812 10.3390/antibiotics12111610 PMC10668804

[B8] Cross K. L. Chirania P. Xiong W. Beall C. J. Elkins J. G. Giannone R. J. . (2018). Insights into the evolution of host association through the isolation and characterization of a novel human periodontal pathobiont, Desulfobulbus oralis. MBio 9:e02061-17. doi: 10.1128/mbio.02061-02017, PMID: 29535201 PMC5850319

[B9] Cui Z. Wang P. Gao W. (2025). Microbial dysbiosis in periodontitis and peri-implantitis: pathogenesis, immune responses, and therapeutic. Front. Cell. Infection Microbiol. 15, 1517154. doi: 10.3389/fcimb.2025.1517154, PMID: 40007610 PMC11850578

[B11] Diaz P. Gonzalo E. Villagra L. J. G. Miegimolle B. Suarez M. J. (2022). What is the prevalence of peri-implantitis? A systematic review and meta-analysis. BMC Oral. Health 22, 449. doi: 10.1186/s12903-022-02493-8, PMID: 36261829 PMC9583568

[B10] Di Spirito F. Giordano F. Di Palo M. P. D’Ambrosio F. Scognamiglio B. Sangiovanni G. . (2024). Microbiota of peri-implant healthy tissues, peri-implant mucositis, and peri-implantitis: A comprehensive review. Microorganisms 12, 1137. doi: 10.3390/microorganisms12061137, PMID: 38930519 PMC11205430

[B12] Douglas G. M. Maffei V. J. Zaneveld J. R. Yurgel S. N. Brown J. R. Taylor C. M. . (2020). PICRUSt2 for prediction of metagenome functions. Nat. Biotechnol. 38, 685–688. doi: 10.1038/s41587-020-0548-6, PMID: 32483366 PMC7365738

[B14] Faveri M. Figueiredo L. C. Shibli J. A. Pérez-Chaparro P. J. Feres M. (2014). “ Microbiological diversity of peri-implantitis biofilms,” in Biofilm-based Healthcare-associated Infections: Volume I (Cham: Springer), 85–96. 10.1007/978-3-319-11038-7_5 25366222

[B15] Fernandes G. V. O. Mosley G. A. Ross W. Dagher A. Martins B. G. D. S. Fernandes J. C. H. (2024). Revisiting Socransky’s complexes: a review suggesting updated new bacterial clusters (GF-MoR Complexes) for periodontal and peri-implant diseases and conditions. Microorganisms 12, 2214. doi: 10.3390/microorganisms12112214, PMID: 39597602 PMC11596145

[B16] Galarraga-Vinueza M. E. Pagni S. Finkelman M. Schoenbaum T. Chambrone L. (2025). Prevalence, incidence, systemic, behavioral, and patient-related risk factors and indicators for peri-implant diseases: an AO/AAP systematic review and meta-analysis. J. periodontol. 96, 587–633. doi: 10.1002/JPER.24-0154, PMID: 40489307 PMC12273760

[B17] Giok K. C. Veettil S. K. Menon R. K. (2024). Risk factors for Peri-implantitis: An umbrella review of meta-analyses of observational studies and assessment of biases. J. dentist 146, 105065. doi: 10.1016/j.jdent.2024.105065, PMID: 38762079

[B18] Joshi A. A. Szafrański S. P. Steglich M. Yang I. Qu T. Schaefer-Dreyer P. . (2025). Integrative microbiome-and metatranscriptome-based analyses reveal diagnostic biomarkers for peri-implantitis. NPJ Biofilms Microbiomes 11, 175. doi: 10.1038/s41522-025-00807-6, PMID: 40858628 PMC12381052

[B19] Kim H.-J. Ahn D.-H. Yu Y. Han H. Kim S. Y. Joo J.-Y. . (2022). Microbial profiling of peri-implantitis compared to the periodontal microbiota in health and disease using 16S rRNA sequencing. J. Periodontal Implant Sci. 53, 69. doi: 10.5051/jpis.2202080104, PMID: 36468472 PMC9943702

[B20] Kushkevych I. Coufalová M. Vítězová M. Rittmann S. K.-M. (2020). Sulfate-reducing bacteria of the oral cavity and their relation with periodontitis—recent advances. J. Clin. Med. 9, 2347. doi: 10.3390/jcm9082347, PMID: 32717883 PMC7464432

[B21] Lafaurie G. I. Castillo D. M. Iniesta M. Sanz M. Gómez L. A. Castillo Y. . (2023). Differential analysis of culturable and unculturable subgingival target microorganisms according to the stages of periodontitis. Clin. Oral. Investig 27, 3029–3043. doi: 10.1007/s00784-023-04907-5, PMID: 36806930 PMC10264511

[B22] Sinjab K. Sawant S. Ou A. Fenno J. C. Wang H. L. Kumar P. (2024). Impact of surface characteristics on the peri-implant microbiome in health and disease. J. periodontol 95, 244–255. doi: 10.1002/JPER.23-0205, PMID: 37665015 PMC10909931

[B23] Song L. Feng Z. Zhou Q. Wu X. Zhang L. Sun Y. . (2024). Metagenomic analysis of healthy and diseased peri-implant microbiome under different periodontal conditions: a cross-sectional study. BMC Oral. Health 24, 105. doi: 10.1186/s12903-023-03442-9, PMID: 38233815 PMC10795403

[B24] Yu P. S. Tu C. C. Wara-aswapati N. Wang C. Y. Tu Y. K. Hou H. H. . (2024). Microbiome of periodontitis and peri-implantitis before and after therapy: Long-read 16S rRNA gene amplicon sequencing. J. Periodontal Res. 59, 657–668. doi: 10.1111/jre.13269, PMID: 38718089

