# Supplementary material for: Peri-implantitis biofilm from explanted implants in Korean patients: microbial and functional profiling
Source: Front Cell Infect Microbiol. 2026 Feb 6;16:1768841. doi: 10.3389/fcimb.2026.1768841 (PMC12920513; doi:10.3389/fcimb.2026.1768841)
Supplement: Supplementary file 1 [file DataSheet1.pdf]

Supplementary Table S1. Sensitivity analysis: Differential abundance between non-smoking individuals

Genus-level differential abundance analysis comparing healthy controls (n=22, all non-smokers) and peri-implantitis cases (n=4, confirmed non-smokers). Only genera with raw $p < 0.05$ are shown. Statistical significance was assessed using Mann-Whitney U test with Benjamini-Hochberg FDR correction. Bold rows indicate FDR-adjusted significance ($q < 0.05$). Beta diversity PERMANOVA: R^2=0.177, p=0.001. Alpha diversity showed significant differences in Shannon (p=0.048) and Simpson (p=0.040) indices. The small sample size (n=4) limits statistical power; results should be interpreted as exploratory findings.

**Genus**	**Healthy (n=22)**	**Peri-implantitis**	**Statistical Significance**	
	Mean ± SD	Mean ± SD	P-value	FDR q value
Pyramidobacter	0.10 ± 0.49	8.29 ± 9.28	2.94×10^{-4}	**0.009**
Porphyromonas	1.21 ± 2.52	7.71 ± 5.95	9.36×10^{-4}	**0.014**
Phocaeicola	0.01 ± 0.04	4.13 ± 7.69	2.85×10^{-3}	**0.024**
Rothia	8.59 ± 9.34	0.78 ± 0.91	3.61×10^{-3}	**0.024**
Corynebacterium	8.79 ± 7.22	0.79 ± 1.58	3.97×10^{-3}	**0.024**
**Bacillus**	0.63 ± 0.96	0.04 ± 0.07	0.01	0.052
**Neisseria**	9.56 ± 7.21	2.17 ± 3.39	0.017	0.074
**Tannerella**	0.73 ± 1.86	13.07 ± 10.39	0.02	0.076
**Treponema**	0.35 ± 0.56	4.45 ± 3.54	0.024	0.081
**Capnocytophaga**	7.98 ± 4.56	2.19 ± 4.28	0.043	0.128

Bold rows indicate statistical significance at FDR-adjusted $q < 0.05$.

Values represent mean relative abundance (%) ± standard deviation.

Supplementary Table S2. Complete genus-level differential abundance analysis

Complete results of Mann-Whitney U test comparing all detected genera (n=182) between peri-implantitis (n=19) and healthy control (n=22) groups. Mean relative abundance (%), Log2 fold change (peri-implantitis/healthy), raw p-values, and FDR-adjusted q-values are shown. Genera are ranked by raw p-value. Bold entries indicate FDR-adjusted significance ($q < 0.05$). Statistical analysis used Benjamini-Hochberg false discovery rate correction. After FDR correction, 63 of 182 genera remained significant at $q < 0.05$.

Supplementary Table S3. LEfSe analysis results

Complete Linear Discriminant Analysis Effect Size (LEfSe) results identifying differentially abundant genera between peri-implantitis (n=19) and healthy control (n=22) groups. The analysis was performed using Kruskal-Wallis test (alpha = 0.05) followed by Linear Discriminant Analysis with LDA score threshold = 2.0. A total of 68 genera showed significant differences (raw $p < 0.05$). All 68 features remained significant after Benjamini-Hochberg FDR correction (FDR $q < 0.05$). Positive LDA scores indicate enrichment in peri-implantitis group; negative scores indicate enrichment in healthy controls. Genera are ranked by raw p-value.

Supplementary Table S4. Multiple testing correction summary

Summary of statistical significance before and after multiple testing correction across all analyses. Complete dataset (n=182 genera), abundant genera subset (n=70, >0.1% mean abundance), LEfSe analysis (n=68 features), and sensitivity analysis (non-smokers only, n=10 nominally significant genera) are shown. Benjamini-Hochberg FDR method was applied as the primary correction method.

Analysis	Uncorrected ($p < 0.05$)	FDR-corrected ($q < 0.05$)	Bonferroni ($p < 0.05$)
Main analysis – All genera (n=182)	68	63	37
Main analysis – Abundant genera (n=70)	55	55	37
LEfSe analysis	68	68	-
Sensitivity analysis (non-smokers)	10	5	-
