# Supplementary material for: Peri-implantitis biofilm from explanted implants in Korean patients: microbial and functional profiling
Source: Front Cell Infect Microbiol. 2026 Feb 6;16:1768841. doi: 10.3389/fcimb.2026.1768841 (PMC12920513; doi:10.3389/fcimb.2026.1768841)
Supplement: Supplementary file 4 [file DataSheet4.pdf]

Supplementary Figure Legends

Supplementary Figure S1. Sensitivity analysis comparing non-smoking individuals only.

Microbial community analysis restricted to non-smoking participants to assess robustness of findings to smoking confounding (Healthy: n=22, all non-smokers vs Peri-implantitis: n=4, confirmed non-smokers). (A) Principal coordinates analysis (PCoA) based on Bray-Curtis distance showing beta diversity patterns. Despite limited sample size, clear clustering separation persists between groups (PERMANOVA: $R^2=0.177$, p=0.001***). Ellipses represent 95% confidence intervals. (B) Alpha diversity comparison between groups. Box plots show median (center line), interquartile range (box), and individual data points. Shannon index (p=0.048*) and Simpson index (p=0.039*) showed significant differences despite reduced sample size. (C) Top 10 differentially abundant genera ranked by Log2 fold change. Blue bars indicate genera enriched in healthy controls; red bars indicate genera enriched in peri-implantitis. Key pathogenic genera (Pyramidobacter, Porphyromonas, Phocaeicola, Tannerella, Treponema) remained significantly enriched after FDR correction. Statistical significance: Mann-Whitney U test with Benjamini-Hochberg FDR correction. *p<0.05, **p<0.01, ***p<0.001 (FDR-adjusted). Complete statistical results are provided in Supplementary Table S1. The reduced effect size compared to the full dataset ($R^2=0.177$ vs 0.596) indicates that demographic factors including smoking contribute to microbiome variation, but core disease-associated signals persist.

Supplementary Figure S2. Study design and workflow.

Comprehensive flowchart illustrating the complete study design, sample collection, laboratory processing, bioinformatics pipeline, and statistical analyses. Samples were obtained from the Apple Tree Oral Biobank, comprising explanted implant surface biofilms from peri-implantitis cases (n=19; age: 62.3±11.5 years; 8F/11M; smoking: 4 non/9 smokers/6 unknown) and subgingival plaque from healthy controls (n=22; age: 28.5±4.3 years; all female; all non-smokers). DNA extraction was performed using LaboPass™ Bacteria Genomic DNA Isolation Kit. The V3-V4 region of 16S rRNA gene was amplified using primers 341F/805R and sequenced on Illumina MiSeq platform (paired-end 2×300 bp). The DADA2 pipeline (v1.30.0) was applied for quality filtering (250/200 bp truncation, EE>2 removal), error correction, denoising, paired-end merging, chimera removal, and ASV generation (2,997 ASVs). Two samples with <10,000 reads were excluded. Rarefaction was performed at 24,639 reads minimum depth. Taxonomic assignment was conducted using SILVA database v138.1 with 50% bootstrap confidence. Statistical analyses included alpha diversity (Shannon, Simpson, PD whole tree), beta diversity (Bray-Curtis distance with PERMANOVA), differential abundance testing (Mann-Whitney U test with FDR correction), LEfSe analysis (LDA score >2.0), functional prediction (PICRUSt2 with NSTI <0.15), and sensitivity analysis restricted to non-smokers (Healthy n=22 vs Peri-implantitis n=4). Final sample size: n=41 (Peri-implantitis n=19, Healthy n=22). Study approved by IRB of Apple Tree Medical Foundation (ATDH-2024-0006). Funding: Korea Disease Control and Prevention Agency (KDCA, 2024ER050701) and Korea Technology and Information Promotion Agency for SMEs (TIPA, 00487164).

Supplementary Figure S3. Microbial composition of individual samples (top 15 genera).

Stacked bar graphs showing the relative abundance of the top 15 genera in each sample. Left panel shows the peri-implantitis group (n=19), and right panel shows the healthy control group (n=22). The red dashed line indicates the boundary between the two groups.

Supplementary Figure S4. Heatmap analysis of the top 30 genera.

Heatmap showing the relative abundance of the top 30 genera in the peri-implantitis group (red bar) and healthy control group (blue bar). The color scale represents Z-score normalized relative abundance, with red indicating high abundance and blue indicating low abundance. Rows and columns were ordered by hierarchical clustering.

Supplementary Figure S5. Genus-level differential abundance analysis.

Significantly differentially abundant genera between the peri-implantitis and healthy control groups ($p < 0.05$). Bar graphs represent Log2 fold change (peri-implantitis/healthy control). Positive values (red bars) indicate genera increased in the peri-implantitis group, while negative values (blue bars) indicate genera increased in the healthy control group. PI, Peri-implantitis; H, Healthy.
