# Supplementary figures and images for: Peri-implantitis biofilm from explanted implants in Korean patients: microbial and functional profiling
Source: Front Cell Infect Microbiol. 2026 Feb 6;16:1768841. doi: 10.3389/fcimb.2026.1768841 (PMC12920513; doi:10.3389/fcimb.2026.1768841)

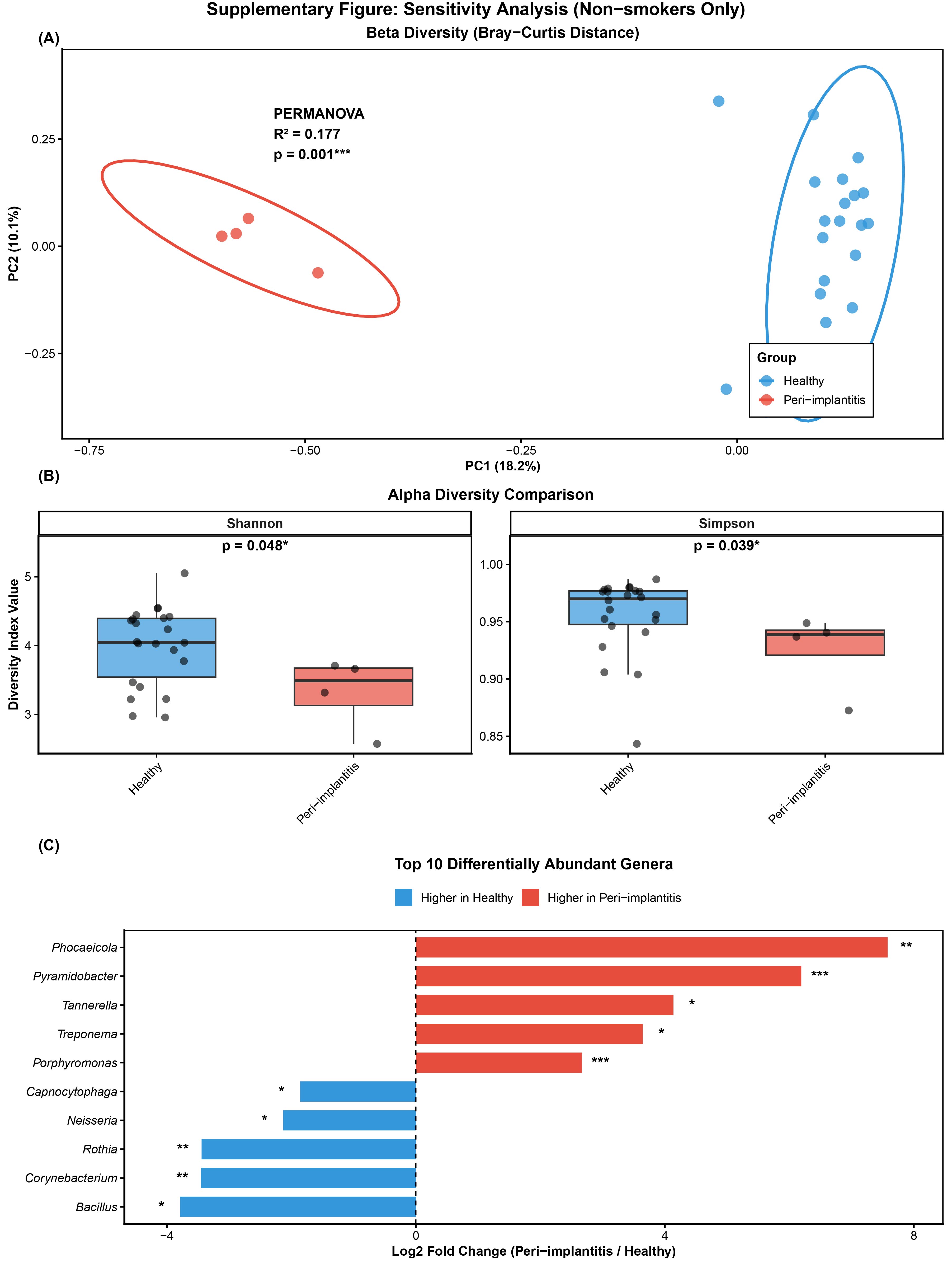

Supplement: Supplementary file 5 [file Image1.jpeg]

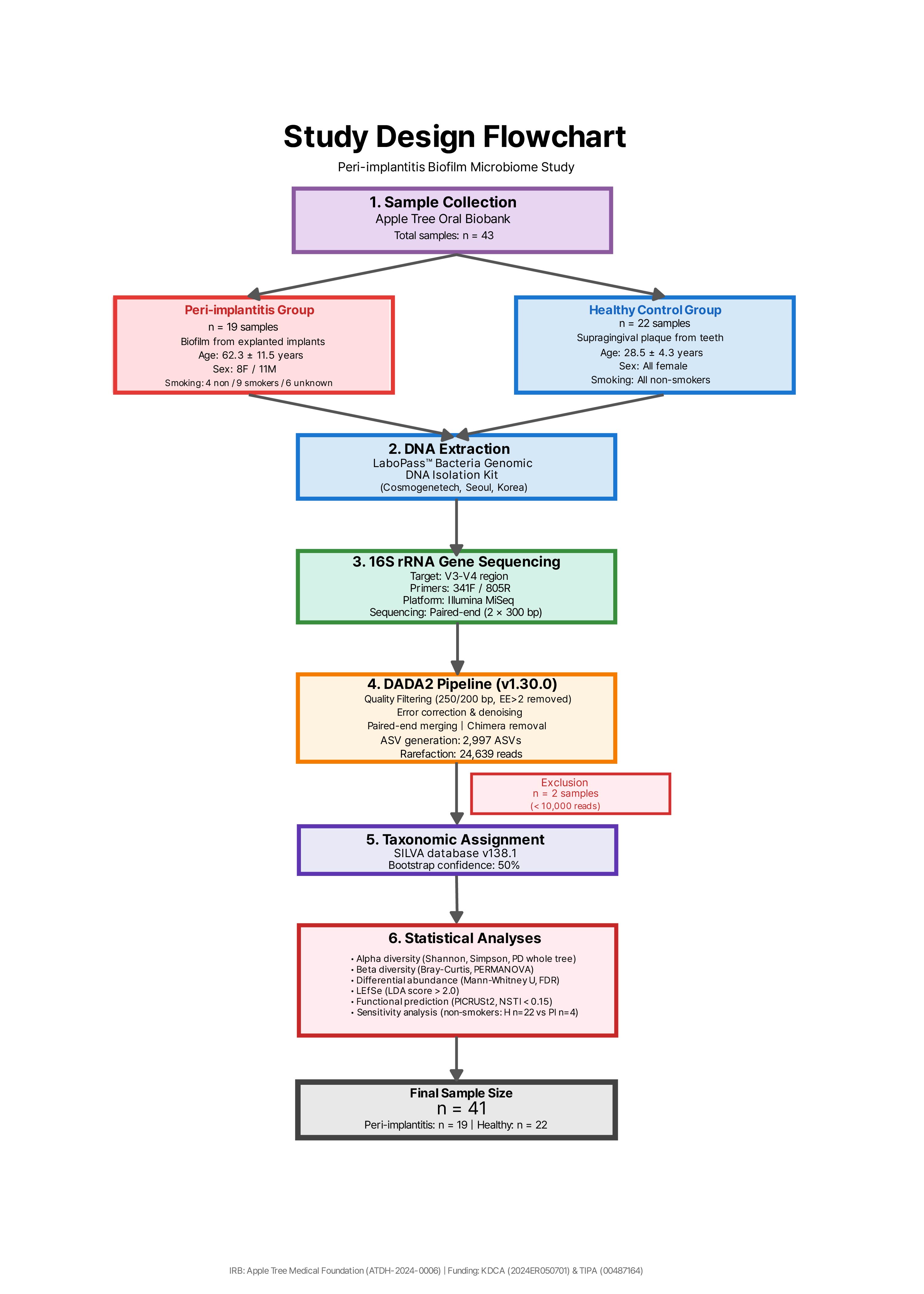

Supplement: Supplementary file 6 [file Image2.jpeg]

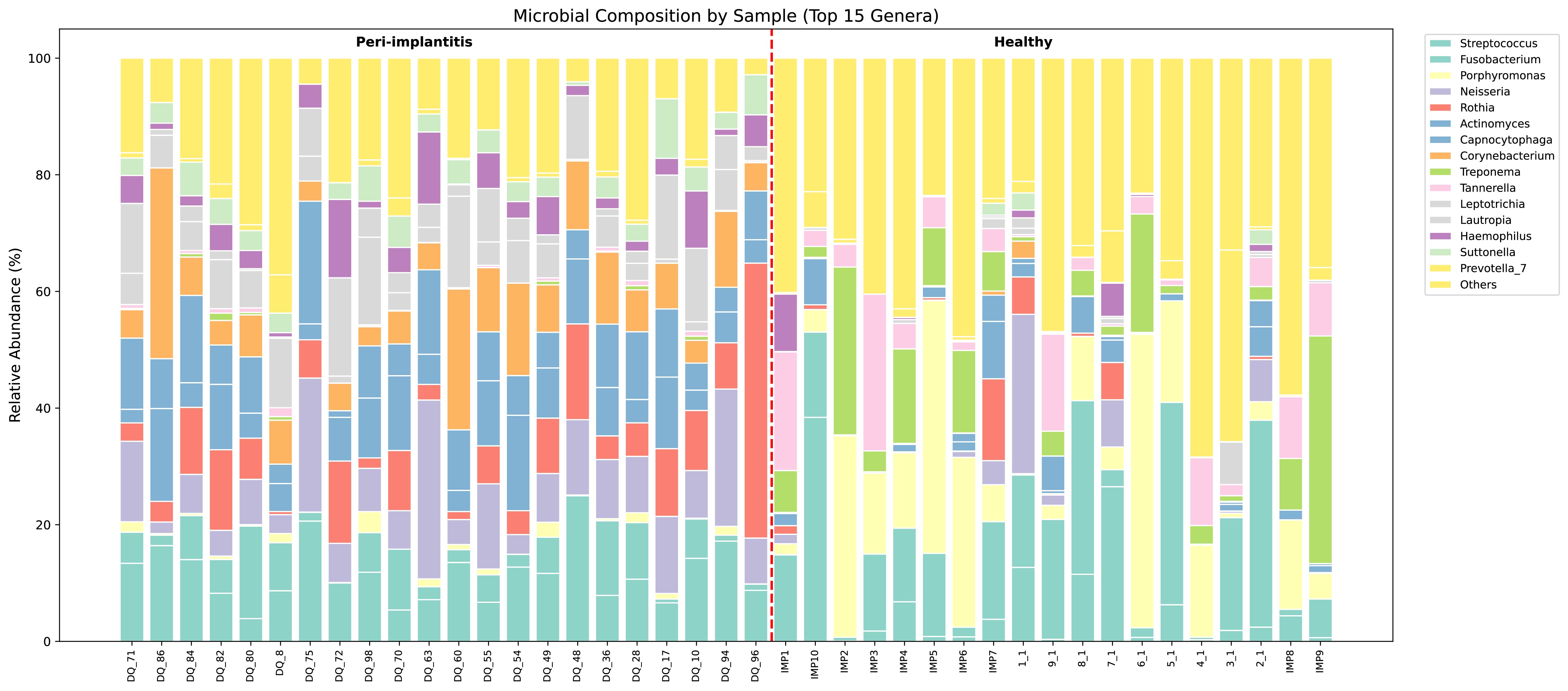

Supplement: Supplementary file 7 [file Image3.jpeg]

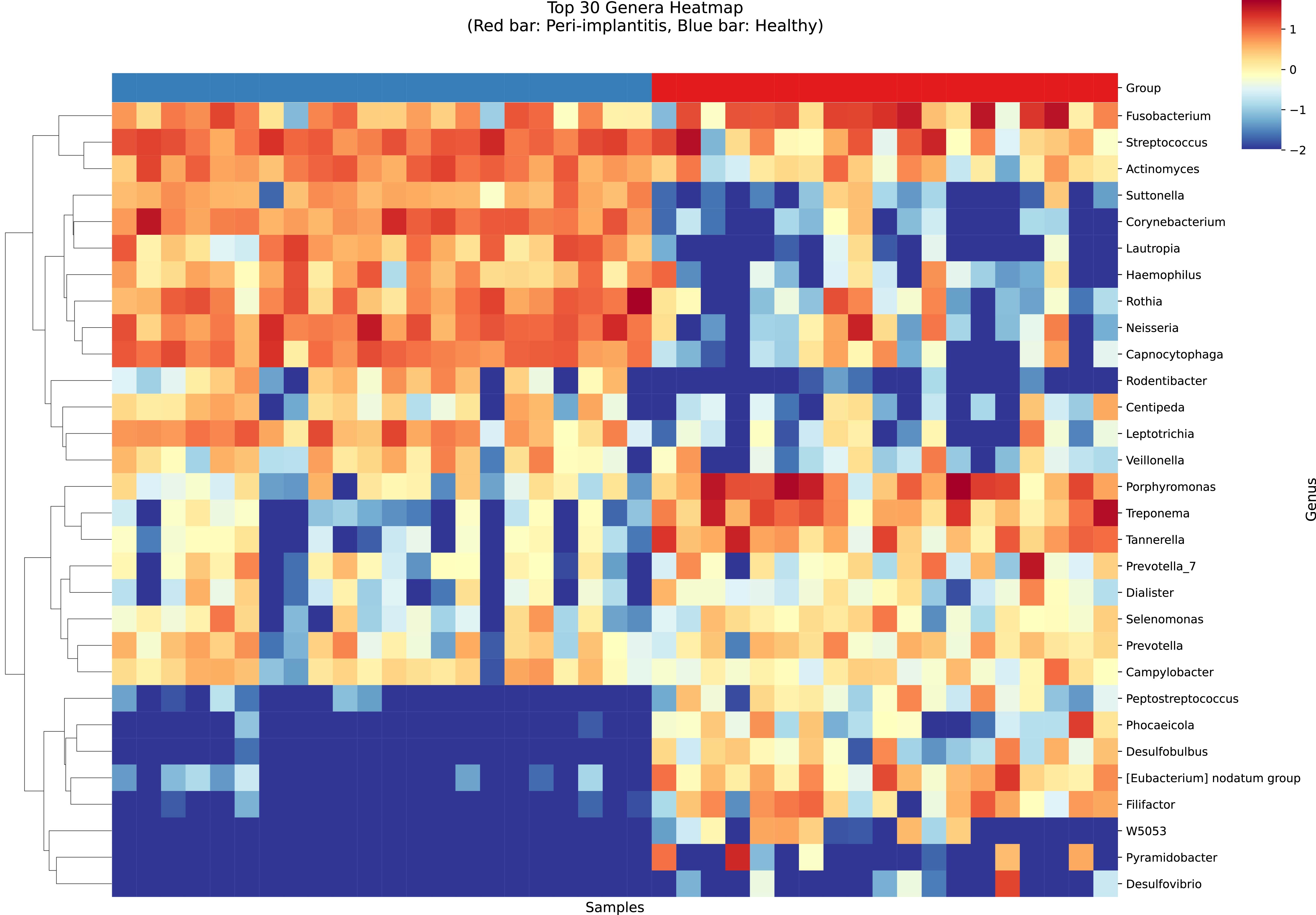

Supplement: Supplementary file 8 [file Image4.jpeg]

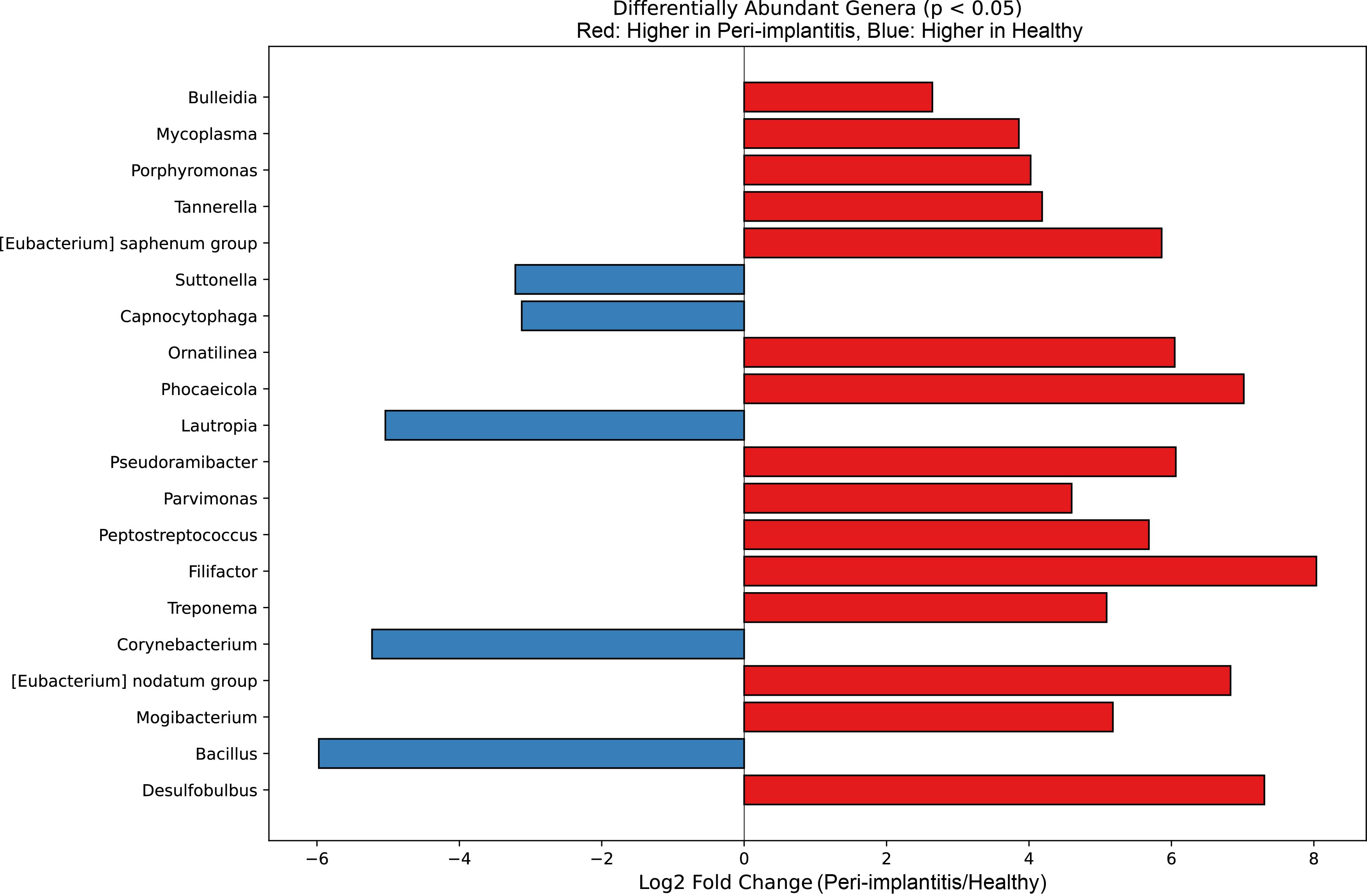

Supplement: Supplementary file 9 [file Image5.jpeg]
